# Diets and environments of late pleistocene pygmy and Columbian mammoths: Isotopic evidence from Southern California

**DOI:** 10.1371/journal.pone.0338674

**Published:** 2026-01-07

**Authors:** Chance D. Hannold, Yang Wang, Xiaoming Wang, Regan Dunn, Jonathan Hoffman

**Affiliations:** 1 Department of Earth, Ocean & Atmospheric Science, Florida State University and National High Magnetic Field Laboratory, Tallahassee, Florida, United States of America; 2 Department of Earth Sciences, University of Southern California, Los Angeles, California, United States of America; 3 Department of Vertebrate Paleontology, Natural History Museum of Los Angeles County, Los Angeles, California, United States of America; 4 La Brea Tar Pits and Museum, Natural History Museums of Los Angeles County, Los Angeles, California, United States of America; 5 Santa Barbara Museum of Natural History, Santa Barbara, California, United States of America; Senckenberg Gesellschaft fur Naturforschung, GERMANY

## Abstract

Pygmy mammoths (*Mammuthus exilis*) and Columbian mammoths (*Mammuthus columbi*) coexisted on the island of Santarosae (now the Northern Channel Islands of California) until the Late Pleistocene megafaunal extinctions, but the ecology of these mammoths is not yet well explored. In this study, we reconstructed the diets and environments of Late Pleistocene pygmy and Columbian mammoths using stable isotopes in tooth enamel samples from the Northern Channel Islands and Rancho La Brea. The enamel δ^13^C values indicate that these mammoths primarily consumed C_3_ vegetation. However, a few individuals consumed significant amounts of C_4_ plants, CAM plants, or water-stressed woody C_3_ plants. The mean diet-δ^13^C value for mainland mammoths (−24.2 ± 1.4‰) is about 2‰ higher than that of island mammoths (−26.4 ± 1.9‰), suggesting that most mainland mammoths consumed either water-stressed C_3_ vegetation, or some C_4_ and/or CAM plants. Reconstructed δ^18^O values of paleo-water from the mainland are generally lower than the mean δ^18^O values of modern precipitation in Southern California, suggesting conditions were wetter and/or cooler than today. Reconstructed δ^18^O values of paleo-water from the islands are more similar to modern precipitation. δ^13^C-based estimates of mean annual precipitation range from 159 to 1407 mm/yr on the islands and from 28 to 387 mm/yr on the mainland. However, consumption of small amounts of C_4_ and/or CAM plants may have resulted in an underestimation of precipitation for the mainland. Radiometric dating of additional fossils from both localities will help clarify the links between climate change and mammoth evolution and extinction in the region.

## 1. Introduction

Pygmy mammoths (*Mammuthus exilis*) are thought to have evolved from Columbian mammoth (*Mammuthus columbi*) populations through insular dwarfism driven by limited resources, competition, and/or predation on the island (known as Foster’s rule or the island rule) [1–5]. Columbian mammoths cohabitated with pygmy mammoths on Santarosae (now the Northern Channel Islands of California, USA, also known as the Channel Islands National Park), but were lower in abundance and restricted to the lower terraces on the island [6]. No specimens of *M. exilis* have been found on the mainland [6], implying that pygmy mammoths were confined to the island. The youngest radiocarbon date—ca. 12,700 cal yr BP (recalibrated using OxCal v.4.4 with IntCal20) [6–8]—associated with pygmy mammoths suggests that this species went extinct around the same time as the Late Pleistocene megafaunal extinctions at the La Brea Tar Pits in Los Angeles, California, USA, around ca. 12.9 ka [9]. The primary driver of these extinctions remains heavily debated [9–18].

Earliest human arrival on Santarosae is radiocarbon dated to ca. 12,900 cal yr BP [7,8,19,20]. However, there is no evidence of humans hunting or butchering pygmy mammoths [21]. Rapid global warming and deglaciation also occurred around this time [22–23]. During the transition to the Holocene, sea level rise inundated lowlands of Santarosae, leaving only the high points (which became the modern Northern Channel Islands, or NCI) subaerially exposed [18,24]. Vegetation also shifted around this time, with conifer forest being replaced by coastal sage scrub, grasslands, and pine stands around 11,800 cal yr BP [25–26]. The close timing of these events makes it difficult to determine a primary extinction mechanism for southern California mammoths. Given the close timing of the extinctions of *M. exilis* (restricted to the NCI) and *M. columbi* (present on the NCI and the mainland), NCI and Rancho La Brea (RLB) mammoths may have shared a primary extinction driver. However, this assumption may be flawed if the niches of the mammoths differ dramatically from the mainland to the islands.

Previous stable isotope work on *M. exilis* (some of which may have been *M. columbi*) had a limited sample size (8 individuals) and a single outlier indicative of mixed feeding [27]. Published works analyzing diet through microwear suggest that pygmy and Columbian mammoths had differing diets, with pygmy mammoths having more attrition-dominated wear suggesting a diet of softer vegetation (such as leaves) and having a smaller dietary range than Columbian mammoths [28–29]. However, stable isotopes have the benefit of reflecting diet throughout tooth growth [30–33] rather than just at the time of death [28]. Previous stable isotope work on *M. columbi* from RLB using dentin [34] and bone collagen [35] yielded anomalously high δ^13^C values (possibly the result of diagenesis) and no data (due to low collagen content), respectively. Enamel is more diagenetically resistant than dentin and bone [36] and is thus more reliable for paleoecological reconstructions [29,37–41].

In this study, we analyzed the carbon and oxygen isotope compositions of more than 200 tooth enamel samples from 34 individual mammoths representing *M. exilis* from the NCI and mainland *M. columbi* from RLB and coastal Santa Barbara in southern California. The data are used to reconstruct diets and environmental conditions to explore the similarities and differences between insular and mainland mammoths. The results are compared with previously published enamel isotope data for *M. columbi* across southern North America to clarify environmental differences between the southern California and other North American mammoth populations during the Late Pleistocene.

### 1.1. Carbon isotopes in plants and mammals

Carbon isotope ratios (δ^13^C) in plants reflect atmospheric δ^13^C, photosynthetic pathway (C_3_, C_4_, or CAM), and environmental factors such as rainfall amount, and amount of canopy coverage [42–46]. C_3_ plants (trees, most shrubs, forbs, and cool-season grasses) typically have δ^13^C values ranging from −37‰ to −23‰, with a mean of about −27‰ [46–47]. Some drought tolerant C_3_ genera can have δ^13^C values as high as −20‰ under severe water stress [46,48–50]. C_4_ plants (mostly warm-season grasses) typically have higher δ^13^C values between −18 and −9‰, with a mean value of −13‰ [43,47,51]. CAM plants (succulents and some epiphytes) have δ^13^C values ranging from −26‰ to −10‰—intermediate between C_3_ and C_4_ plants—and typically inhabit water-limited environments [51–54]. These values in plants shift in response to atmospheric δ^13^C values [55]. These plant δ^13^C values are incorporated into the structural carbonate of herbivore bone and dental tissue [43,56] with an isotopic enrichment [57–59]. The mean enamel-diet enrichment (Ɛ_enamel-diet_) for modern elephants—considered as modern analogs of mammoths [60]—is approximately +14.1‰ [57,59]. Some researchers have proposed using body mass (BM)-based estimates to calculate the enrichment factor [58], which would result in a higher estimate for *M. columbi* (+15.1‰) [61] and a lower estimate for *M. exilis* (+13.8‰). However, modern ponies have higher enamel-diet enrichments than modern horses [59], so whether the relationship is applicable for dwarfed species is unclear.

### 1.2. Oxygen isotopes in meteoric water and mammals

Oxygen isotope ratios (δ^18^O) in meteoric water are controlled by climatic conditions, including moisture source, air temperature (temperature effect), distance the vapor has traveled inland away from the moisture source (continental effect), elevation of land below the vapor (altitude effect), and amount of rainfall (amount effect) [62–63]. Surface water (such as rain puddles, streams, lakes/ponds, and springs) provides the source of drinking water for animals. The oxygen isotope ratios of drinking water, food, and air are incorporated into the structural carbonate and phosphate of mammalian bone and dental tissue [64–67]. Enamel and bone apatite δ^18^O values reflect primarily drinking water for drought intolerant species (or obligate drinkers) and dietary water (i.e., leaf water) for drought tolerant species (or non-obligate drinkers) [68–70]. For obligate drinkers, their bioapatite δ^18^O values are strongly correlated to the δ^18^O values of local meteoric water [64,65,69,71,72] and these modern relationships may be used to reconstruct the δ^18^O values of local paleo-water [73–74].

### 1.3. Modern conditions in southern California

**Fig 1:**
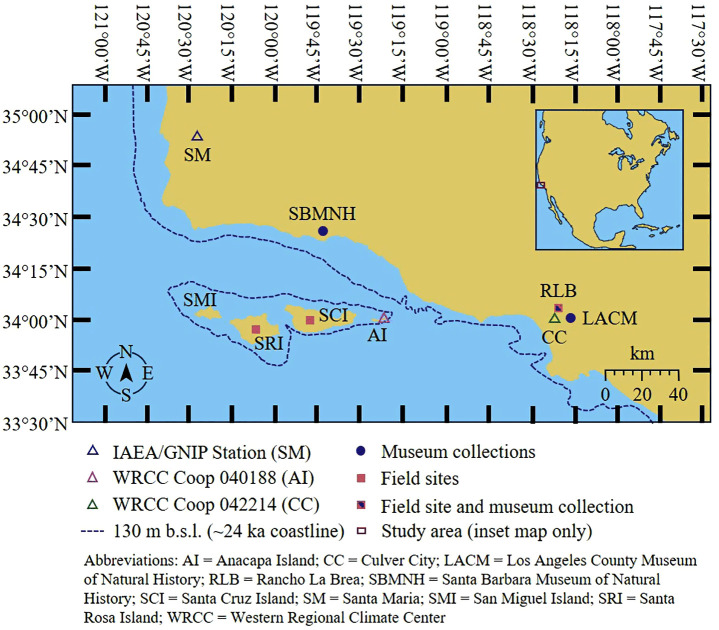
Northern Channel Islands and southern California mammoth sites. The sites and museum collections from which mammoth fossils were recovered, and stations from which meteorological data were sourced, are labeled on the map. The approximate paleo-coastlines of Santarosae and mainland California with a lowstand sea level of ~130 m below modern sea level is approximated using Google Earth depth measurements.

Study sites include two localities: the Northern Channel Islands (NCI) and Rancho La Brea (RLB, also known as the La Brea Tar Pits and Museum). The NCI are an east-west oriented chain of islands off the coast of California (Fig 1) [5,75,76]. These islands are largely a series of marine terraces covered by more recent eolian sands and alluvium deposits, with mammoth fossils present in both marine and terrestrial deposits [24,75,77]. RLB is a site that has active asphalt seeps and fossil-rich asphalt deposits that continue to be excavated [78–79]. The plants in both localities predominantly use C_3_ photosynthesis due to the Mediterranean climate of the region [25,44,45,53,80]. Notably, native C_4_ plants are absent from the Channel Islands [44,45,81]. CAM plants on the other hand are present on the Channel Islands and in southern California (such as *Dudleya* and *Opuntia*) [82–83].

**Fig 2:**
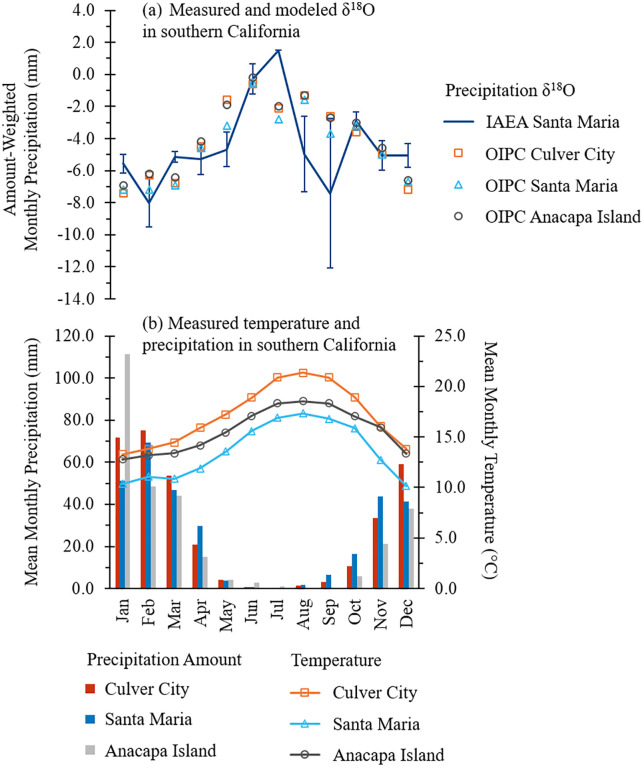
Modern rainfall amount and oxygen isotope composition in southern California. (a) The modern mean amount-weighted monthly precipitation δ^18^O values for the period of 1962-1976 recorded at IAEA/GNIP station in Santa Maria, California, are represented by the line, with vertical error bars indicating 1 standard deviation from the mean [84]. Precipitation δ^18^O values modeled by the Online Isotopes in Precipitation Calculator (OIPC) for Culver City, Santa Maria, and Anacapa Island—based on modern elevation and latitude—are shown as points [85–87]. (b) Modern mean monthly precipitation amounts are depicted by bars and modern monthly temperatures are represented by lines. Precipitation and temperature data for Culver City and Anacapa Island are from Western Regional Climate Center COOP stations; data for Santa Maria are from the IAEA/GNIP station.

The recent mean annual precipitation (MAP) near NCI and RLB are 292.4 mm and 334.0 mm (using data from Anacapa Island and Culver City), respectively (Western Regional Climate Center, http://www.wrcc.dri.edu). The recent mean annual temperature (MAT) near NCI and RLB are 15.6 ± 2.2°C and 17.1 ± 3.0°C, respectively (Western Regional Climate Center, http://www.wrcc.dri.edu). The nearest IAEA/GNIP station is in Santa Maria, California. Santa Maria’s MAP is 312.4 mm [84], which is comparable to the MAP observed at the NCI and RLB. Although the temperature in Santa Maria (13.6 ± 2.8°C) is slightly cooler than those observed at NCI and RLB, the precipitation δ^18^O values estimated using the Online Isotopes in Precipitation Calculator (OIPC) [85–87] are similar across these localities (Fig 2a). Precipitation in Santa Maria exhibits higher amount-weighted δ^18^O values during the dry summer months and lower values in winter (Fig 2) [84]. The long-term annual mean δ^18^O value of precipitation from 1962 to 1976 at the IAEA station in Santa Maria is −4.21 ± 1.03‰, while the corresponding long-term annual amount-weighted mean oxygen isotope ratio of precipitation (δ^18^O_weighted.precip_) for the same period is −5.94 ± 1.48‰ [84], reflecting the influence of heavier rainfall with more negative isotopic values from the non-summer months. The annual mean δ^18^O values of modern precipitation calculated using the OIPC (−5.5‰ and −5.4‰, respectively) are essentially identical for the NCI (latitude: 34.00°, longitude: 119.88°W, altitude: 140.5 m a.s.l.) and RLB (latitude: 34.06°, longitude: 119.88°W, altitude: 140.5 m a.s.l.), reflecting the close similarity in climatic conditions between these localities. While the annual δ^18^O_weighted.precip_ values differ slightly (~0.5‰) between Santa Maria and NCI/RLB, the seasonal pattern appears to be similar (Fig 2).

### 1.4. Radiometric dates for santarosae and rancho la brea

Available radiocarbon (^14^C) and uranium/thorium (U/Th) dates for bones and teeth of pygmy and Columbian mammoths from the NCI show a range of 68,000 ± 4000 U/Th yr BP [6,88,89] to ca. 12,600 cal yr BP [28]. However, indirect radiometric dates obtained from U/Th dating of underlying corals and ^14^C dating of overlying land snails, suggest that the age of one pygmy mammoth tusk from Santa Rosa Island is between ca. 120 ka and ca. 40 ka [77]. Another pygmy mammoth tusk is constrained by U/Th dating of nearby corals to be no younger than ca. 80 ka [77]. While it is possible that mammoth colonization of the NCI occurred even earlier as suggested by Muhs et al. (2015), most dated NCI mammoth fossils are much younger than the estimated dates of these two tusks. Reported calibrated radiocarbon dates for RLB megafauna range from ca. 62,500 cal yr BP to 11,500 cal yr BP [9,90,91]. Pit 9, from which most of the RLB mammoths in this study have been excavated, has yielded radiocarbon dates ranging from 62,000–14,000 cal yr BP [90]. Based on these previously reported radiometric dates, the estimated ages are ~ 68–13 ka for NCI samples [6,28,88,89] and ~62–12 ka for RLB samples [9,90,91].

### 1.5. Pleistocene conditions in the channel Islands and Southern California

During the Pleistocene glacial periods, the seafloor between and surrounding the current NCI was exposed due to glacially-induced low sea level, connecting the current NCI as one long island called Santarosae [20,24]. Multi-proxy eustatic sea level reconstructions [92] indicate that global sea levels were on average ~90 m below modern sea level (b.s.l.) between ~68–12 ka, with a lowstand sea level of 130 m b.s.l. and a highstand sea level of 51 m b.s.l. These are not significantly different from older estimates of between 74 m and 125 m lower sea level for the Late Pleistocene NCI [5,93]. Combining current mean elevation (140.5 m above sea level for the NCI, 57 m above sea level for RLB) [76] and lower sea level estimates [92], elevation on Santarosae is estimated to be approximately 232 m above sea level (a.s.l.) (ranging from 204 m a.s.l. to 271 m a.s.l.) and elevation at RLB is estimated to be approximately 149 m a.s.l. (ranging from 108 m to 187 m a.s.l.) during the last glacial period.

During the last glacial period, the expansion of continental ice sheets significantly lowered sea levels and increased the heavy oxygen isotope (^18^O) content of the seawater – the primary source of moisture for precipitation on land. The oxygen isotope ratio of seawater (δ^18^O_SW_) decreases by 0.009‰ per meter of sea level increase [92,94]. Based on this relationship and the sea level reconstructions for ~68–12 ka, the global mean δ^18^O_SW_ value would be 0.81‰ (relative to V-SMOW). The global mean δ^18^O_SW_ value would be lower during the highstand (0.46‰) and higher during the lowstand (1.17‰). These estimates are consistent with previous studies which suggest that global ocean water was enriched in ^18^O by an average of 1.0 ± 0.1‰ during the Last Glacial Maximum (LGM), and by approximately 0.5–1.0‰ during the period from 70 ka to 12 ka, relative to today [94–97].

During the lowstand, the distance between the nearest points of Santarosae and the mainland would have been ~7 km (Fig 1). This is certainly a swimmable distance for modern elephants and should have been for Columbian mammoths [98–100]. Even the modern distance of ~20 km to Anacapa Island today is still swimmable by modern elephants [77,98], and so dispersals or crossings during the highstand—across a distance of ~12 km from the mainland to Anacapa Island—are not impossible. However, it is arguable whether the Pygmy mammoths would have been able to return to the mainland due to morphological changes associated with dwarfism (i.e., shorter trunks, reduced skeletal pneumatization), which may have reduced their swimming capabilities [98,101,102].

Pollen records from a marine core in the Santa Barbara Channel indicate that open coniferous forests—dominated by juniper/cypress and pine—flourished on the mainland and probably on NCI before onset of Bølling Allerød warming at ~14.7 ka. After this time, juniper/cypress began a steep decline and were replaced by oak, chaparral taxa and coastal sage assemblages dominated by members of the Asteraceae [103]. A macrofloral assemblage from Santa Cruz Island [104], spanning 17,020 cal yr BP to 15,160 cal yr BP [105–106], contains diverse mesic pine and cypress taxa, along with other woody species (e.g., manzanita) that currently grow in coastal Northern California. Younger pollen records from Santa Rosa Island reveal a dominance of non-arboreal, coastal sage species and grasses, indicating that a relatively rapid vegetation turnover occurred on the islands from 15 to 12 ka [25]. This vegetation shift coincides with an increase in human occupation sites and wildfire activity between 13,000 and 11,000 cal yr BP [18,107,108].

The last glacial period was cooler than pre-industrial climate, with a global peak cooling of 4–6°C below pre-industrial temperature during the LGM [109–110]. However, this cooler climate was not without perturbations; large, rapid warming events (interstadials) oscillated with cooling events (stadials) throughout this period [111]. Northern hemisphere interstadials generally had relatively warmer temperatures (though still cooler than modern) and wetter conditions (with dry summers and wet winters), while stadials generally had colder temperatures with drier conditions overall [112–117].

## 2. Materials and methods

### 2.1. Enamel collection

Samples of enamel powder (n = 217) were collected by drilling mammoth teeth (N = 34) using a handheld rotary tool with a diamond tipped burr. No permits were required for the described study, which complied with all relevant regulations. Pygmy mammoth (*Mammuthus exilis*) teeth (N = 20, n = 145) were sampled from the collections of the Santa Barbara Museum of Natural History (SBMNH) and the Los Angeles County Museum of Natural History (LACM). The provenance of ORR 11 and ORR 12 is uncertain (these may have been from either Santa Rosa Island or Santa Cruz Island). All other NCI pygmy mammoths were collected from alluvial sediments on the northern shore of Santa Rosa Island. The distinction between islands may be irrelevant given all NCI represent highlands of the continuous, large island of Santarosae during the age of these mammoths due to lower sea level during this period. Some of the individual teeth (LACM/CIT 177, LACM/CIT 178, LACM/CIT 179, LACM/CIT 209, LACM/CIT 907, LACM/CIT 68714) sampled by Parry (2020) from LACM were resampled in this study to compare isotopic values and confirm that methods are comparable between these studies. Columbian mammoth (*Mammuthus columbi*) teeth (N = 14, n = 72) were sampled from the collections of the La Brea Tar Pits Museum (RLB) and SBMNH. The sole Columbian mammoth tooth sampled at SBMNH (ORR 6) was collected from the coastal Santa Barbara (SB) area roughly 100 years ago, although it is unclear whether the specimen is from the mainland or the NCI. Species identifications for island mammoths were from previous identifications based on tooth size and enamel plate width; however, the sampled mammoths fall within the overlapping ranges of *M. columbi* and *M. exilis* teeth [118]. Due to the limited specimens available for sampling in museum collections, available teeth were sampled regardless of tooth position. While we tentatively assume each tooth represents a unique individual mammoth—and some must indeed come from distinct individuals based on different collection localities (e.g., different canyons on the NCI) and/or duplication of the same element type (e.g., upper left 3rd molars) (S1 Table)—we cannot be certain that all sampled teeth represent unique individuals.

**Fig 3:**
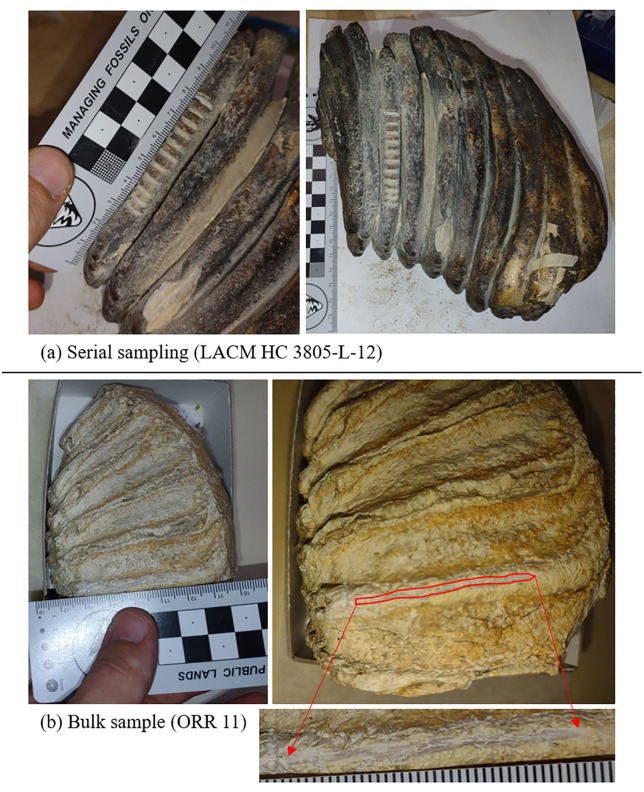
Drilling strategies for (a) serial and (b) bulk tooth samples. Photos are taken with a reference ruler (cm) as a scale. (a) Serial samples of LACM HC 3805-L-12 are taken perpendicular to the growth axis and measure from the occlusal most point of the measured plate. (b) A bulk sample of ORR 11 is taken parallel to the growth axis.

Outer enamel surfaces were cleaned of any glue or surface coating when necessary and abraded using the drill prior to sample collection following the common practice [119–120]. In some cases, cementum was removed via drill abrasion to access the enamel surface for sampling. Drill bits were wiped clean or replaced between removal of outer surfaces and sample collection. Serial samples of enamel were collected by drilling perpendicular to the growth axis at several points along the tooth (Fig 3a). Care was taken to avoid collecting dentine while drilling by lengthening drill lines when necessary [119]. Given the estimated enamel growth rates for mammoths of 13–14 mm in crown height per year [121], each drilled serial sample (~2.0 to 2.5 mm wide) represents a mean of the isotopic values for about 2 months and the separation of drill cuts is about the same amount of time. Depth of drill cuts was approximately 1–2 mm. Bulk samples were collected by drilling parallel to the growth axis (Fig 3b). Drill cut depths for bulk samples were the same and width was approximately 2 mm. Lengths of bulk drill cuts were 20–30 mm on average, though longer cuts were taken when possible (Fig 3b).

### 2.2. Ages of sampled mammoths

The ages of our tooth enamel samples were constrained to the last glacial period [111,114,116,122] by a series of radiometric dates on fossil bones and teeth and associated charcoals reported from the fossil localities [6,9,28,88–91]. Based on these previously reported radiometric dates, the estimated ages are ~ 68–13 ka for our NCI samples [6,28,88,89] and ~ 62–12 ka for our RLB samples [9,90,91]. Direct dating of the sampled specimens was not performed to minimize destructive sampling of collection specimens and due to the high failure rate (up to 78%) for extraction of collagen from tar pit fossils [123].

### 2.3. Chemical treatment and isotopic analysis

Enamel samples were treated following a commonly used procedure [56,119,120,124–127]. Samples were treated with 5% sodium hypochlorite in a fume hood at room temperature overnight to remove organic matter, cleaned with deionized water, followed by treatment with 1 M acetic acid in a fume hood at room temperature overnight to remove non-structural carbonate. The treated samples (containing only hydroxyapatite) were then cleaned with deionized water and freeze-dried. The dried samples (2–3 mg each) were weighed into reaction vials and capped with rubber septa. The vials were then loaded into the sample block of a Finnigan GasBench II Auto-carbonate device.

For each batch of samples, multiple sets of carbonate standards—selected to bracket the expected range of sample values—were included at the beginning, after every 10–15 samples, and at the end of the sequence. Each set of standards consisted of triplicate vials of each individual standard (one of each standard to serve as an accuracy check, two of each standard averaged to serve as calibration standards). The vials were then flushed with ultra-pure helium to remove air, after which 3–4 drops of 100% phosphoric acid were injected into each vial with standard and 6–8 drops were injected into each vial with sample to react with the powders for approximately 72 hours at 25 ºC. The resulting carbon dioxide was introduced via the GasBench II in a continuous helium stream into the Finnigan MAT Delta Plus XP stable isotope ratio mass spectrometer (IRMS), located in the Stable Isotope Lab at the National High Magnetic Field Laboratory (NHMFL), for analysis of carbon and oxygen isotope ratios (δ^13^C and δ^18^O).

The intra-lab calibration standards (ROY-cc: δ^13^C = 0.67‰ and δ^18^O = −12.02‰; MB-cc: δ^13^C = −10.5‰ and δ^18^O = −3.14‰; and MERK: δ^13^C = −35.5‰ and δ^18^O = −16.2‰) are homogeneous carbonate powders—calibrated to the international standard Vienna Peedee Belemnite (V-PDB) using IAEA reference materials NBS 18 (δ^13^C = −5.01‰ and δ^18^O = −23.01‰) and NBS 19 (δ^13^C = +1.95‰ and δ^18^O = −2.20‰)—that were used to calibrate measured sample values to the V-PDB scale. Specifically, the measured and expected values of each calibration standard were used to construct a 3-point calibration curve via linear regression. The resulting calibration equation was then applied to the measured values of accuracy check standards and samples to obtain the calibrated values, reported as δ^13^C and δ^18^O relative to V-PDB. Analytical precision and accuracy were evaluated using the accuracy check standards. Precision was calculated as one standard deviation (σ) from the mean of the calibrated values for each accuracy check standard, while accuracy was assessed as the absolute difference (AD) between the calibrated mean and the expected value for each standard. The calculated accuracies and precisions for the different accuracy check standards were then averaged to obtain the mean accuracy and precision for the dataset. Mean accuracy and precision for carbonate δ^13^C measurements were 0.1‰ (AD) and 0.1‰ (1σ), respectively, based on repeated analyses of lab standards. Mean accuracy and precision for carbonate δ^18^O measurements were 0.1‰ (AD) and 0.1‰ (1σ). All enamel carbonate results are reported in the standard δ notation as δ^13^C and δ^18^O values in permil (‰) relative to V-PDB (S2 Table).

### 2.4. Diagenesis

The teeth we sampled were all morphologically well-preserved, showing no visible signs of alteration. The enamel color matched that of modern enamel [128]. Calcium carbonate weights (wt. CaCO_3_) for samples were estimated from measured ion intensities (mV of the m/z 44 peaks) using the regression equation derived from the relationship between measured ion intensities and weights of the carbonate standards. Calcium carbonate weight percentages (wt% CaCO_3_) were then calculated by dividing these estimated carbonate weights by the measured weights for enamel samples after chemical pretreatment. The estimated mean wt% CaCO_3_ for samples (6.7 ± 0.9%) was high relative to the range of 3.2–4.6% observed in modern mammalian enamel [124,129] because these values do not account for weight loss during chemical pretreatment. Although we did not measure the sample weights before chemical treatments in this study, our pretreatment procedure typically results in weight losses of ~20% in Miocene enamel and ~50% in modern enamel. Assuming a mean weight loss of 35% during chemical pretreatment—representing the average of losses observed for Miocene and modern enamel—the mean wt% CaCO_3_ estimated using pretreated powder weights for these mammoth fossils would correspond to wt% CaCO_3_ of ~4.4% in the untreated enamel, consistent with observations in modern enamel. The estimated mean wt% CaCO_3_ does not differ between localities (NCI = 6.5 ± 0.9%; RLB = 6.9 ± 0.9%), despite the expected higher diagenetic susceptibility of RLB samples due to burial with natural asphalt and the high failure rate of collagen extraction in mammoth material from the site [35]. Additionally, the lack of correlation of enamel carbon isotope ratios (δ^13^C_en_) and oxygen isotope ratios (δ^18^O_en_) with estimated wt% CaCO_3_ (R^2^ values = 0.0923 and 0.0938, respectively) would be unexpected if significant amounts of diagenetic carbonate were present during analysis [128]. Moreover, if diagenetic carbonate had been analyzed, we would expect the correlation between δ^18^O_en_ and wt% CaCO_3_ to be much stronger than that between δ^13^C_en_ and wt% CaCO_3_ due to the differential susceptibility to diagenetic alteration of these isotopic systems in enamel [36]. While we cannot fully exclude the possibility of alteration—especially for RLB samples, given the previous issues with mammoth materials from this site [34,35]—the sampled mammoth enamel is tentatively interpreted as unaltered, based on their morphological integrity, the similarity in mean wt% CaCO_3_ between localities, and the lack of correlations between estimated wt% CaCO_3_ and δ^13^C_en_ or δ^18^O_en_ values.

### 2.5. Reconstruction of modern-equivalent diet δ^13^C

The modern-equivalent diet carbon isotope ratios (δ^13^C_diet:meq_) of mammoths were reconstructed from the enamel δ^13^C values using the following equation (Table 1; S3 Table) [55,57,130]:

**Table 1:** Locality means of carbon and oxygen isotope ratios.

Subdivision	Means (in ‰)				Standard Deviation (in ‰)	
	δ^13^C_en_ (vs. V-PDB)	δ^13^C_diet:meq_ (vs. V-PDB)	δ^18^O_en_ (vs. V-PDB)	δ^18^O_water:meq_ (vs. V-SMOW)	δ^13^C_diet:meq_	δ^18^O_water:meq_
RLB (*M. columbi*)	−9.2	−24.2	−4.4	−7.1	1.4	0.9
SB (*M. columbi*)	−12.5	−27.5	−1.4	−3.9	N/A	N/A
NCI (*M. exilis*)	−11.4	−26.4	−2.9	−5.5	1.9	1.0
All samples	−10.6	−25.6	−3.4	−6.1	2.0	1.3

Species: *Mammuthus columbi*; *Mammuthus exilis.*

Locality: Northern Channel Islands, NCI; coastal Santa Barbara, SB; Rancho La Brea, RLB.


$$\delta^{\text{13}}{\text{C}}_{\text{diet}:\text{meq}}\;=\;\delta^{\text{13}}{\text{C}}_{\text{en}}-\epsilon_{\text{enamel}-\text{diet}}\;+\;(\delta^{\text{13}}{\text{C}}_{\text{atm}:\text{modern}}\;-\;\delta^{\text{13}}{\text{C}}_{\text{atm}:\text{study}})$$ (1)

where δ^13^C_diet:meq_ is the carbon isotope composition of dietary vegetation adjusted to modern atmospheric carbon isotope ratios, δ^13^C_en_ is the carbon isotope composition of enamel carbonate, Ɛ_enamel-diet_ is the carbon isotope enrichment between enamel carbonate and diet (+14.1‰), δ^13^C_atm:modern_ is the carbon isotope composition of modern atmospheric carbon dioxide, and δ^13^C_atm:study_ is the carbon isotope composition of atmospheric carbon dioxide during the age range of the fossils analyzed. Alternative dietary values (δ^13^C_diet:meq-BM_) were also reconstructed for comparison, using enrichment factors for Columbian mammoths [61] and for pygmy mammoths calculated using the empirical relationship between body mass (BM) [131] and enrichment factor given in Tejada-Lara et al. (2018) (S1 Fig; S3 Table). While we prefer the modern elephant value of Ɛ_enamel-diet_ given that the relationship with body mass has been shown not to be consistent in modern species [59] and dwarfed species (e.g.,., ponies, pygmy hippos) appear to have higher Ɛ_enamel-diet_ values than their ancestral species (e.g., horses, hippos) [59] rather than lower values as would be predicted by body mass-dependent estimates [58], the difference in δ^13^C_diet:meq_ values between methods—while reducing the gap between locations by 1.3‰—would not change the interpretations of environmental conditions and/or dietary preference for either location. The carbon isotope composition of atmospheric carbon dioxide from 1990 to 2000 C.E. ranged between −8.20‰ and –7.60‰, with a mean of −7.92‰ [132]. Since much of the published work establishing C_3_ and C_4_ carbon isotope compositions is from this period, a value of approximately −7.92‰ should be appropriate for δ^13^C_atm:modern_ in reconstructions. The mean reconstructed δ^13^C value of the atmospheric CO_2_ (δ^13^C_atm:study_) based on high resolution benthic foraminifera [55] was −6.98‰ between 0.06 Ma and 0.01 Ma. As such, a correction of −0.94‰ was applied to NCI/SB and RLB paleo-diet/paleo-vegetation δ^13^C values to estimate a modern equivalent. These values were compared to modern vegetation to narrow down possible diet (S4 Table).

### 2.6. Reconstruction of paleo-water δ^18^O

Enamel oxygen isotope composition depends on various physiological factors that differ between species, but oxygen isotope composition strongly correlates with local drinking water isotopic composition in obligate drinkers [64,72]. Ayliffe et al. (1992) found the following relationship in elephants (which are obligate drinkers and which we consider a modern analog for mammoths):


$$\delta^{\text{18}}{\text{O}}_{\text{p}}\;=\;\text{0}.\text{94}\;(+\;\text{0}.\text{10})\;\times \;\delta^{\text{18}}{\text{O}}_{\text{water}}\;+\;\text{23}.\text{30}\;(\pm \;\text{0}.\text{7})$$ (2)

where δ^18^O_water_ is the oxygen isotope composition of the environmental water relative to V-SMOW and δ^18^O_p_ is the oxygen isotope composition of bone or enamel phosphate relative to V-SMOW. By substituting the relationship between δ^18^O values relative to V-SMOW in carbonate and phosphate in modern mammals [133] into Eq. 2 and converting δ^18^O_c_ from relative to V-SMOW to relative to V-PDB [134], the relationship between the oxygen isotope compositions of environmental water and proboscidean enamel carbonate (assuming the fossils are not substantially altered) was simplified as:


$$\delta^{\text{18}}{\text{O}}_{\text{water}}\;=\;\text{1}.\text{0748}\;(+\;\text{0}.\text{1143})\;\times \;\delta^{\text{1}\text{8}}{\text{O}}_{\text{c}}\;–\;\text{1}.\text{5940}\;(\pm \;\text{0}.\text{1762})$$ (3)

where δ^18^O_water_ is the oxygen isotope composition of the environmental water relative to V-SMOW and δ^18^O_c_ is the oxygen isotope composition of bone or enamel carbonate relative to V-PDB. Enamel oxygen isotope ratios (δ^18^O_en_) (Table 1; S2 Table) were used as δ^18^O_c_ values in this equation to reconstruct the local water isotope ratios (δ^18^O_water_) (S3 Table) relative to V-SMOW. Alternative reconstructed water values (δ^18^O_water, general_) using the general equation for obligate drinkers [72] were also calculated for comparison (S3 Table). Although we favor the species-specific relationships between enamel and water δ^18^O values given the differences observed in these relationships for modern species [69,71,135], this alternative approach resulted in only small shifts (as low as 0.01‰ and only up to 0.23‰) and would not change interpretations if used (S1 Fig). For comparison with modern δ^18^O_weighted.precip_ values, the modern equivalent of local water oxygen isotope ratios (δ^18^O_water:meq_) (Table 1; S3 Table) were calculated by subtracting the estimated paleo-δ^18^O_SW_ value (~0.8‰) between ~68−13 ka from all δ^18^O_water_ values to account for the isotopic difference in the moisture sources (i.e., between Pleistocene seawater and modern seawater). Modern precipitation oxygen isotope data in the study region (Fig 2) were obtained from IAEA/WMO (2023) and are reported as δ^18^O_weighted.precip_ values relative to V-SMOW.

### 2.7. δ^13^C-based reconstruction of mean annual precipitation

Kohn (2010) compiled δ^13^C values of modern C_3_ plants from various ecosystems around the world and found the following relationship:


$$\delta^{\text{13}}\text{C}\;=\;\text{1}.\text{90}\;\times \;{\text{10}}^{-\text{4}\;}\text{ALT}\;-\;\text{5}.\text{61}\;\times \;{\text{log}}_{\text{10}}\;(\text{MAP}\;+\;\text{300})\;-\;\text{0}.\text{0124}\;\text{abs}(\text{LAT})\;-\;\text{10}.\text{29}$$ (4)

where δ^13^C is the carbon isotope composition of C_3_ vegetation, ALT is altitude in m, MAP is mean annual precipitation in mm/yr, and LAT is latitude in degrees.

The above equation was used to estimate MAP from the mean δ^13^C_diet:meq_ estimated for each fossil tooth, along with the latitude (34.00°N for NCI and 34.06°N for RLB) and estimated mean altitudes (232 m a.s.l. for NCI and 149 m a.s.l. for RLB) at each locality. Plant δ^13^C values greater than −23.0‰ either reflect C_3_ plants under severe water stress and restricted to excessively dry regions (i.e., MAP less than 10 mm/yr) or non-C_3_ plants (C_4_ or CAM), and the inclusion of these would lead to an underestimate of MAP using this equation [46]. For this study, the maximum δ^13^C value used for MAP reconstruction is −24.6‰, in order to avoid inclusion of mixed feeders whose diets included a significant portion (17% or more) of C_4_ or CAM plants, which would otherwise result in negative rainfall estimates. However, even this small amount of C_4_ or CAM plant consumption may have resulted in an underestimate of MAP and therefore MAP projections in this study should be taken as lower end estimates. The means of serial samples were used to provide approximate bulk estimates for each serially sampled specimen for use in calculating MAP estimates, allowing for comparison with estimates from bulk samples. While previous studies [136–138] have noted differences in bulk sampled isotopic data and averaged serially sampled isotopic data, dual methodology sampling for one tooth (LACM HC 68190)—for which the bulk yielded δ^13^C and δ^18^O values of −9.4‰ and −2.6‰, respectively, and the mean of serial samples yielded values of −10.0‰ and −2.9‰, respectively—showed an absolute difference in δ^13^C and δ^18^O values of only 0.6‰ and 0.3‰, respectively. While this admittedly does present a difference in values outside of analytical uncertainty, these differences are small relative to the observed mean difference between localities (S2 Fig). Because these differences are not large enough to obscure locality differences, the isotopic difference between sampling methods should not prohibit including all samples for reconstructions regardless of sampling method. MAP estimates derived using δ^13^C_diet:meq-BM_ (MAP_BM_) were also calculated for comparison (S1 Fig; S3 Table), though we favor MAP over MAP_BM_ for the same reasons as discussed for δ^13^C_diet:meq_ and δ^13^C_diet:meq-BM_.

### 2.8. δ^18^O-based reconstruction of temperature and precipitation

Modern relationships between monthly amount-weighted oxygen isotope values of precipitation (δ^18^O_weighted.precip_ [‰ vs. V-SMOW]) and monthly mean air temperature (T [°C]) (Eq. 5) and monthly δ^18^O_weighted.precip_ values and mean monthly precipitation amount (MMP [mm]) (Eq. 6) in Santa Maria from 1962 to 1976 were determined using regression analysis of recorded monthly data from the region [84].


$${\delta \text{1}}^{\text{1}\text{8}}{\text{O}}_{\text{weighted}.\text{precip}}\;=\;\text{0}.\text{4519}\;\times \;\text{T}\;-\;\text{10}.\text{555}\;(\text{R}\;=\;\text{0}.\text{585},\;{\text{R}}^{\text{2}}\;=\;\text{0}.\text{343})$$ (5)

$$\delta^{\text{18}}{\text{O}}_{\text{weighted}.\text{precip}}\;=\;-\text{0}.\text{0605}\;\times \;\text{MMP}\;-\;\text{2}.\text{8413}\;(\text{R}\;=\;\text{0}.\text{731},\;{\text{R}}^{\text{2}}\;=\;\text{0}.\text{534})$$ (6)

Reconstructed δ^18^O_water:meq_ should reflect rainfall-derived bodies of water in obligate drinkers and is generally assumed to be equivalent to δ^18^O_weighted.precip_. However, if a significant proportion of an animal’s drinking water came from evaporated sources such as ponds and lakes, the reconstructed δ^18^O_water:meq_ based on enamel δ^18^O values would be higher than the δ^18^O of local precipitation. This is because evaporation preferentially removes lighter isotopes into the vapor phase, leaving the remaining water enriched in the heavy oxygen isotope ^18^O [139].

The weak coefficient of determination between δ^18^O_weighted.precip_ values and temperature (R^2^ = 0.343) and the moderate coefficient of determination between δ^18^O_weighted.precip_ values and rainfall amount (R^2^ = 0.534) likely reflect the mild seasonal temperature range and relatively low peak rainfall in southern California (relative to more humid regions of the continent) from 1962 to 1976. Temperature and δ^18^O_weighted.precip_ values are moderately positively correlated (R = 0.585) (i.e., less negative δ^18^O_water:meq_ values reflect warmer conditions and more negative δ^18^O_water:meq_ values reflect cooler conditions). Precipitation amount and δ^18^O_weighted.precip_ values are strongly negatively correlated (R = 0.731) (i.e., less negative δ^18^O_water:meq_ reflect drier conditions and more negative δ^18^O_water:meq_ values reflect wetter conditions). Considering the strength of correlations and the weakness of coefficients of determination, differences in δ^18^O_water:meq_ were interpreted qualitatively rather than quantified using Eq. 5 or Eq. 6. It is important to note that these relationships are also dependent on regional climate (including prevailing wind and circulation patterns) and may not hold if the regional climate in southern California differed in the past. However, while the strength of the correlation between δ^18^O_weighted.precip_ values and temperature is weaker for southern California than that for global precipitation δ^18^O values and temperature [140], the positive directionality of the correlation is consistent with global patterns and may still hold even if regional climate differed in the past.

## 3. Results and Interpretation

### 3.1. Stable isotopes, diets, and environments of mammoths from NCI/coastal Santa Barbara

**Fig 4:**
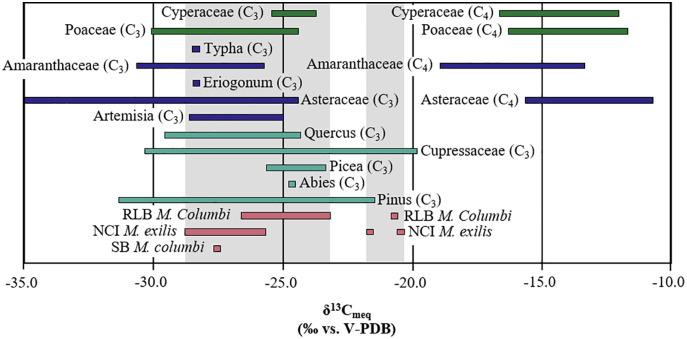
Modern equivalent carbon isotope compositions of plant groups present in Pleistocene southern California and reconstructed diets of sampled mammoths. Dark green bars indicate plant taxa with primarily graminoid growth habits [141–143]. Blue bars indicate taxa with primarily forb to shrub habits [141,142,144–147]. Light green bars indicate taxa with primarily tree growth habits [49,141,148–160]. The light gray shaded zones represent ranges of reconstructed mammoth diets, separated between diets possible with only C_3_ plants and those that require either water-stressed pine and cypress, C_4_, and/or CAM plants. Ranges of mammoths in each locality are indicated by pale violet red bars. Plant groups (families and genera) depicted here were present at relative abundances of 15% or above at some time in the pollen record in southern California [26,103], or were a subdivision of a plant family that did reach that level of abundance.

**Fig 5:**
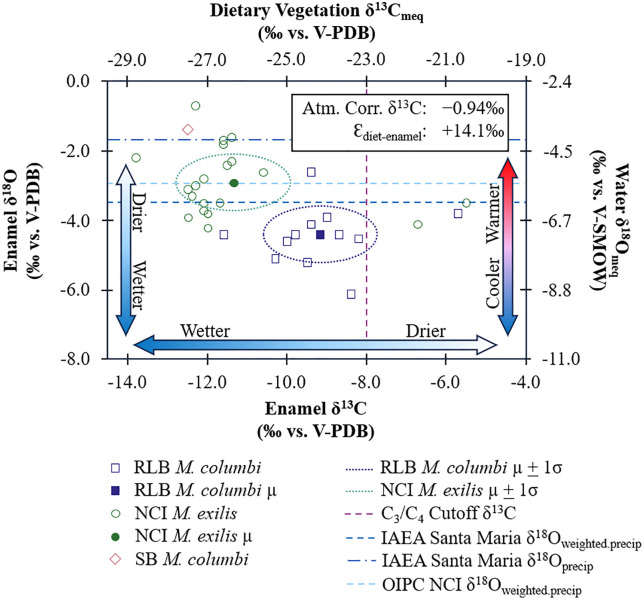
Enamel isotopic compositions of mammoths by location and species. The purple dashed line represents the δ^13^C cutoff between C_3_ and C_4_ vegetation (δ^13^C_meq_ = −23.0‰), or between a pure browsing/typical C_3_ plant diet and mixed C_3_-C_4_ feeding diet expected in fossil enamel assuming an atmospheric correction of −0.94‰ between 0.06 and 0.01 Ma and assuming an enrichment of +14.1‰ between diet and enamel for proboscideans. The dark blue dashed line represents the long-term amount-weighted oxygen isotope ratios for precipitation (δ^18^O_weighted.precip_) measured in Santa Maria from 1962-1976 [84]. The dark blue dash-dot line represents long-term unweighted annual oxygen isotope ratios for precipitation (δ^18^O_precip_) measured in Santa Maria from the same period [84]. The light blue dashed line represents the δ^18^O_weighted.precip_ calculated using the OIPC [85–87] for the NCI. Measured mammoth enamel δ^13^C and δ^18^O values are presented as open symbols separated by species (*Mammuthus columbi* or *Mammuthus exilis*) and location. Localities include Northern Channel Islands (NCI), coastal Santa Barbara (SB), and Rancho La Brea (RLB). The means of serial samples were used as approximate bulk values to include with measured values from bulk samples. Locality means (µ) and one standard deviation (σ) are presented as closed symbols and dotted ovals, respectively. Vertical arrows with gradients represent relative differences in precipitation amount or air temperature inferred from enamel δ^18^O values based on modern relationships between δ^18^O_weighted.precip_ and precipitation amount or δ^18^O_weighted.precip_ and air temperature, respectively, in Santa Maria from 1962-1976. The horizontal arrow with gradient represents relative differences in precipitation amount inferred from enamel δ^13^C values based on modern relationships between plant δ^13^C values and precipitation amount [46].

The mean δ^13^C_en_ and δ^18^O_en_ values of NCI mammoths are −11.4‰ (−12.2‰, −10.6‰; 95% CI) and −2.9‰ (−3.3‰, −2.5‰; 95% CI), respectively (Fig 5; Table 1). The modern equivalent of dietary vegetation would have a mean δ^13^C_diet:meq_ value of −26.4‰ (−27.2‰, −25.6‰; 95% CI) (Figs 4 and 5). Using the alternative BM-based estimate of Ɛ_enamel-diet_ for *M. exilis* produces δ^13^C_diet:meq-BM_ values 0.3‰ higher than δ^13^C_diet:meq_ values (S1 Fig; S3 Table). The reconstructed δ^13^C_diet:meq_ (or δ^13^C_diet: meq-BM_) values for NCI mammoths are well within the δ^13^C range of modern C_3_ plants, except for two individuals (LACM/CIT 178 and ORR 11) that may have consumed a mixture of C_3_ and C_4_ or CAM plants, or fed on water-stressed C_3_ woody plants, as evidenced by their higher δ^13^C values (i.e., δ^13^C_en_ > −6.8‰ or δ^13^C_diet:meq_ > −22‰) (Figs 4 and 5). Notably, Parry (2020) also sampled LACM/CIT 178, one of the two outliers exhibiting higher δ^13^C_en_ values, and obtained identical results within analytical uncertainty. The single SB mammoth (ORR 6) had δ^13^C_en_ and δ^18^O_en_ values of −12.5‰ and −1.4‰, respectively, both of which fall within the range of δ^13^C_en_ and δ^18^O_en_ values observed in NCI mammoths and outside the range of δ^13^C_en_ and δ^18^O_en_ values observed in RLB mammoths (Table 1; Fig 5). The similarity in enamel isotope composition between ORR 6 and the NCI mammoths (Table 1; Fig 5) suggests similar diets (mean difference = 1.1‰) and water sources (mean difference = 1.6‰).

**Fig 6:**
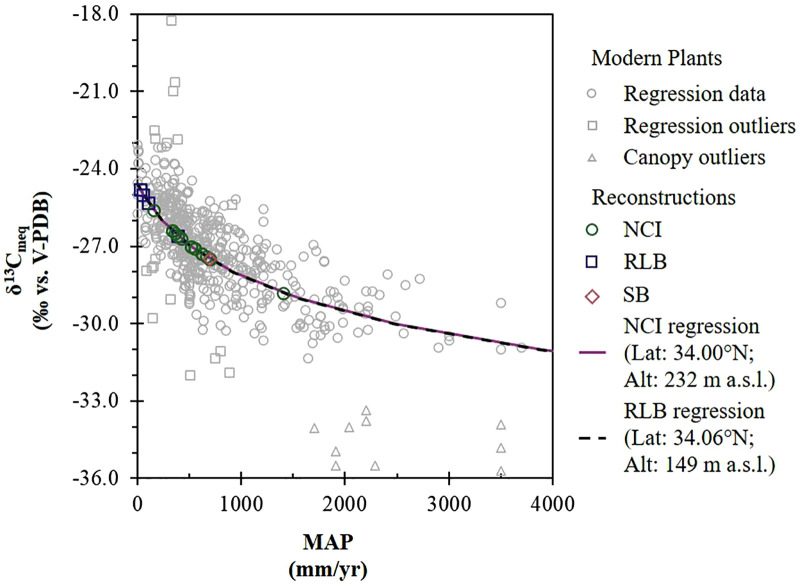
Mean annual precipitation and vegetation carbon isotope ratios. Reconstructed modern equivalent δ^13^C values of dietary vegetation (excluding those above −24.6‰) are plotted in the context of isotopic values for modern vegetation reported in the supplemental material of Kohn (2010) and curve fits to Eq. 4 for each locality.

MAP for the NCI (Fig 6; Table 2) was estimated using the reconstructed δ^13^C_diet:meq_ values (S3 Table), altitude and latitude estimates for ~68−13 ka, and Eq. 4. The estimated MAP for NCI mammoths is between 159 and 1407 mm/yr, with a mean of 544 mm/yr (424 mm/yr, 664 mm/yr; 95% CI). The mean estimated MAP_BM_ for pygmy mammoths is 446 mm/yr (340 mm/yr, 552 mm/yr; 95% CI) (S3 Table). Both estimates are higher than the mean annual rainfall amount recorded on the present-day NCI (292.4 mm/yr) (Western Regional Climate Center, http://www.wrcc.dri.edu), suggesting a wetter climate during that time interval than today. The maximal estimate comes from a pygmy mammoth (ORR 10) that had much lower δ^13^C_en_ than other pygmy mammoths sampled; however, even when this is omitted, the mean MAP estimate of 493 mm/yr (422 mm/yr, 564 mm/yr; 95% CI) is still much higher than the modern MAP of 292.4 mm/yr. The estimated MAP for the SB mammoth is 701 mm/yr, also much higher than modern rainfall in Santa Maria (312.4 mm/yr) [84] or the NCI (292.4 mm/yr) (Western Regional Climate Center, http://www.wrcc.dri.edu). This value falls within the range of MAP for NCI mammoths, even when ORR 10 is omitted.

**Table 2:** Mean annual precipitation (MAP) estimates for mammoth localities.

Subdivision	Mean Annual Precipitation (mm/yr)				
	Median	Mean	Standard Deviation	Minimum	Maximum
RLB (*M. columbi*)	56	121	152	28	387
SB (*M. columbi*)	701	N/A	N/A	N/A	N/A
NCI (*M. exilis*)	532	544	260	159	1407
All samples	468	462	295	28	1407

Species: *Mammuthus columbi*; *Mammuthus exilis*

Locality: Northern Channel Islands, NCI; coastal Santa Barbara, SB; Rancho La Brea, RLB

Reconstructed mean δ^18^O_water:meq_ value for NCI mammoths is −5.5 ± 1.0‰ (Table 1). Comparison with the modern annual mean δ^18^O_weighted.precip_ value (−5.94 ± 1.48‰) recorded at the IAEA station in Santa Maria (Figs 2 and 5) and the OIPC-calculated modern annual δ^18^O_weighted.precip_ value (−5.5‰) [85–87] for the NCI suggests that the NCI experienced a similar climate to modern Santa Maria and the modern NCI. The small difference (0.4‰) can likely be attributed to data scatter and uncertainties in the reconstruction equations.

### 3.2. Stable isotopes, diets, and environments of mammoths from Rancho La Brea

The mean δ^13^C_en_ and δ^18^O_en_ values of RLB mammoths are −9.2‰ (−10.0‰, −8.4‰; 95% CI) and −4.4‰ (−4.8‰, −4.0‰; 95% CI), respectively (Fig 5; Table 1). The reconstructed modern equivalent of dietary vegetation has a mean δ^13^C_diet:meq_ value of −24.2‰ (−25.0‰, −23.4‰; 95% CI) (Figs 4 and 5; Table 1). Using the alternative BM-based estimate of Ɛ_enamel-diet_ for *M. columbi* [61] yields δ^13^C_diet:meq-BM_ values that are 1.0‰ lower than δ^13^C_diet:meq_ values (S1 Fig; S3 Table). These reconstructed modern-equivalent diet-δ^13^C values, while generally higher than those of the NCI mammoths (Fig 4), still fall within the δ^13^C range of C_3_ plants, suggesting C_3_-dominated diets, with one exception (LACM HC 68184). This outlier RLB mammoth (LACM HC 68184) had a high δ^13^C_en_ value of −5.7‰ (Fig 5), corresponding to a reconstructed δ^13^C_diet:meq_ value of −20.7‰ (Fig 5), which suggests either a mixed C_3_-C_4_/CAM diet or a diet consisting of woody C_3_ plants experiencing severe water stress.

MAP for RLB (Fig 6; Table 2) was estimated using the reconstructed δ^13^C_diet:meq_ values (S3 Table), altitude and latitude estimates for ~62−12 ka, and Eq. 4. The estimated MAP for RLB mammoths is between 28 and 387 mm/yr, with a mean of 121 mm/yr (0 mm, 254 mm; 95% CI). Mean estimated MAP_BM_ is 212 mm/yr for RLB (S3 Table). Both MAP and MAP_BM_ estimates are lower than the modern MAP of 334.0 mm/yr in Culver City (Western Regional Climate Center, http://www.wrcc.dri.edu). One RLB Columbian mammoth (LACM HC 68579) had a dietary δ^13^C value indicative of much higher MAP (376 mm/yr) than the mean MAP of all other RLB mammoths (54 mm/yr), possibly coinciding with a wetter period than these other RLB mammoths. Consumption of C_4_ or CAM plants may have caused underestimation of MAP with Eq. 4.

Reconstructed δ^18^O_water:meq_ values from RLB mammoths suggest a water source with a mean δ^18^O value of −7.1‰ (Fig 5; Table 1), which is lower than the mean δ^18^O value of modern precipitation (δ^18^O_weighted.precip_ = −5.94 ± 1.48‰; δ^18^O_precip_ = −4.21 ± 1.03‰) recorded at the IAEA station in Santa Maria (Fig 2) [84]—and also lower than the mean precipitation δ^18^O value of −5.4‰ calculated using the OIPC [85–87] for RLB—suggesting wetter and/or cooler conditions for Late Pleistocene RLB than today at either Santa Maria or RLB. These estimated mean δ^18^O_water:meq_ values are more negative than the mean on Santarosae, likely reflecting temporal differences in the hydroclimate experienced by mammoths at these localities. It is worth noting that two RLB Columbian mammoths (LACM HC 68190 and LACM HC 68579) have δ^13^C_en_ and δ^18^O_en_ values approaching the most negative values of the NCI mammoths (Fig 5), possibly examples of contemporaneous mammoths at the two sites.

### 3.3. Intra-tooth isotope variations in mammoths from NCI/coastal Santa Barbara and Rancho La Brea

**Fig 7:**
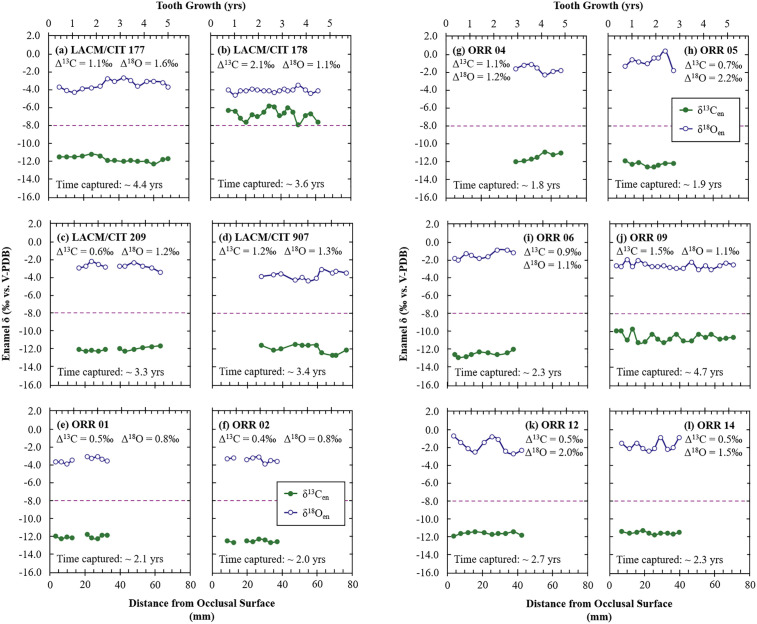
Serial carbon and oxygen isotope compositions of Santarosae/coastal Santa Barbara mammoths from the Museum of Natural History of Los Angeles County collections (a-d) and from the Santa Barbara Museum of Natural History collections (e-l). Enamel δ^13^C and δ^18^O values are plotted against distance along each tooth from the occlusal surface. All mammoths serially sampled from LACM and SBMNH are *M. exilis*, except for ORR 6 which is a tooth from *M. columbi* collected from the coastal area near the Northern Channel Islands. The maximum intra-tooth ranges of δ^13^C_en_ and δ^18^O_en_ values are provided as Δ^13^C and Δ^18^O values. The approximate time captured by samples assumes a growth rate of 14 mm for 1.0 year [121]. The tooth growth axis assumes this growth rate and treats the occlusal surface as the starting point of tooth growth. Note that this starting point does not account for loss of enamel due to wear of the occlusal surface during mastication. The purple dashed lines represent the δ^13^C cutoff (δ^13^C_en_ = −8.0‰) between a pure browsing/typical C_3_ plant diet and mixed C_3_-C_4_ feeding diet expected in fossil enamel assuming an atmospheric correction of −0.94‰ between 0.06 and 0.01 Ma and assuming an enrichment of +14.1‰ between diet and enamel for proboscideans. Gaps in the δ^13^C_en_ and δ^18^O_en_ lines of panels (c), (e), and (f) indicate a missing sampling point for each of these mammoths due to vial septa leaks and air contamination during sample analysis.

**Fig 8:**
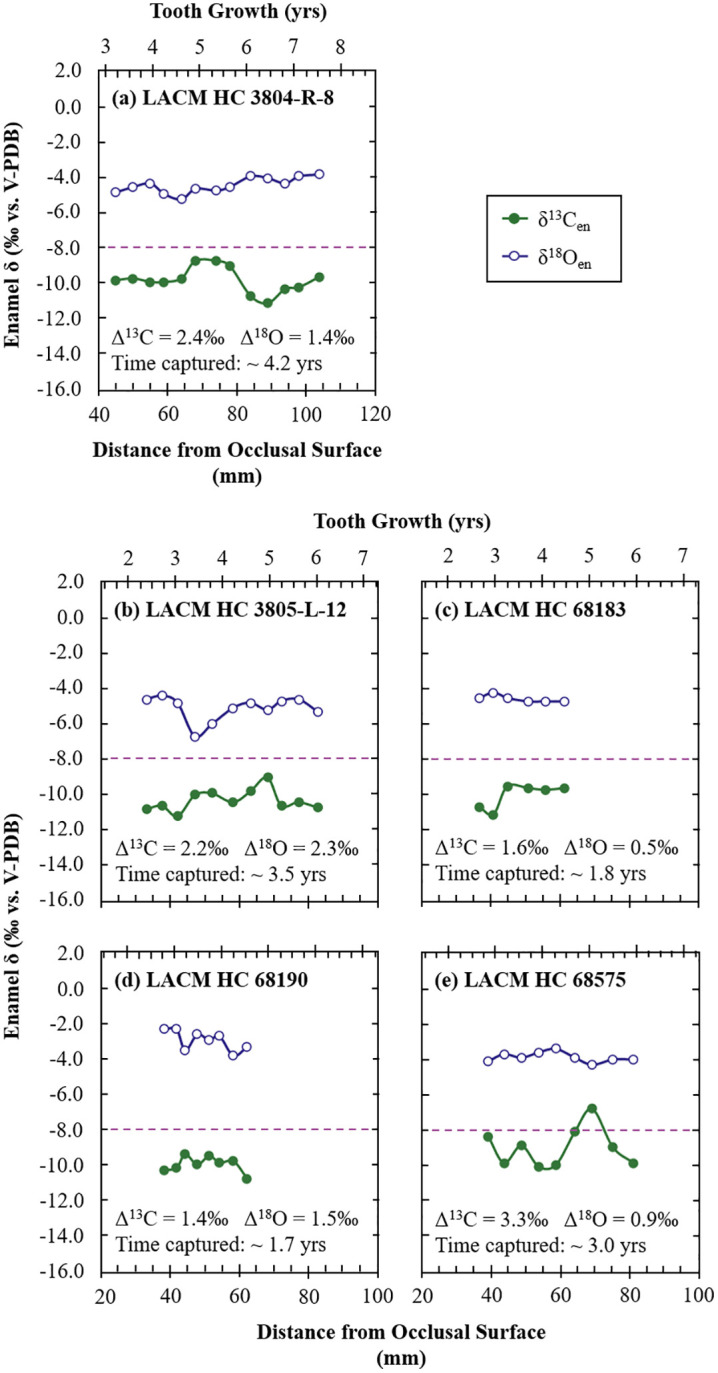
Serial carbon and oxygen isotope compositions of Columbian mammoths from the La Brea Tar Pits and Museum collections. Enamel δ^13^C and δ^18^O values are plotted against distance along each tooth from the occlusal surface. All mammoths serially sampled from RLB are *M. columbi*. The maximum intra-tooth ranges of δ^13^C_en_ and δ^18^O_en_ values are provided as Δ^13^C and Δ^18^O values. The approximate time captured by samples assumes a growth rate of 14 mm for 1.0 year [121]. The tooth growth axis assumes this growth rate and treats the occlusal surface as the starting point of tooth growth. Note that this starting point does not account for loss of enamel due to wear of the occlusal surface during mastication. The purple dashed lines represent the δ^13^C cutoff (δ^13^C_en_ = −8.0‰) between a pure browsing/typical C_3_ plant diet and mixed C_3_-C_4_ feeding diet expected in fossil enamel assuming an atmospheric correction of −0.94‰ between 0.06 and 0.01 Ma and assuming an enrichment of +14.1‰ between diet and enamel for proboscideans.

Most of the serially sampled teeth from the NCI had a small intra-tooth variability in δ^13^C_en_ and δ^18^O_en_ values (Fig 7; S4 Table), with mean ranges of 0.9‰ and 1.4‰, respectively. Serial samples of teeth from RLB show greater variability in δ^13^C_en_ and similar variability in δ^18^O_en_ values (Fig 8) with a mean range of 2.2‰ and 1.3‰, respectively. Patterns within the isotopic composition of these serial samples are evaluated for seasonality assuming that within a year of enamel growth [121], the lowest δ^18^O_en_ value represents winter and the highest value represents summer [30,84]. The δ^13^C_en_ values are then interpreted in this seasonal framework to determine if there is a seasonality in diet. Unfortunately, the loss of a data point in LACM/CIT 209, ORR 1, and ORR 2 (Figs 7c, 7e, and 7f) due to vial or septa leaks makes discussion of possible patterns in intra-tooth ranges of isotopic compositions of these specific teeth difficult.

Some of the intra-tooth isotope profiles exhibit sinusoidal patterns across ~13–14 mm of tooth length, consistent with annual cycles, suggesting seasonal variation in meteoric water (Figs 7a-7b, 7i–7l-7l, 8a-8b, and 8d-8e) and/or diet (such as in ORR 9). For RLB mammoths, these sinusoidal patterns are often superimposed on longer-term trends (Figs 8a and 8d) or interrupted by abrupt shifts (Fig 8b), likely reflecting the greater range of mobility on the mainland. Assuming mammoths behaved similarly to elephants, movements may have included seasonal migration, range expansion, natal dispersal, or nomadism [161]. Seasonal migration tends to flatten the isotopic signals [30,162], whereas other movement types can produce more irregular patterns (e.g., shifts in mean values reflecting change in locality/environment) or variability over longer time scales. Migration is assumed to have been limited to within the islands for *M. exilis*—given the inference that pygmy mammoths could not effectively cross the channel to the mainland [98], whereas *M. columbi* likely undertook broader movements, including potential channel crossings. Although this assumption may not hold during the LGM when lowered sea levels greatly reduced the channel distance (Fig 1), the slightly larger amplitudes in the δ^18^O_en_ sinusoidal patterns in NCI mammoths (range: ± 0.3 to ± 1.0‰, mean: ± 0.6‰) compared to RLB mammoths (range: ± 0.3 to ± 0.4‰) support the assumption that seasonal migration was more common or extensive among mainland mammoths. Other teeth show relatively flat or irregular patterns in δ^18^O_en_ values (Figs 7h and 8c) or δ^13^C_en_ values (Figs 7a, 7g-7h, 7k-7l, and 8c), likely reflecting isotopic damping [32,163,164], seasonal migration [30,162], and/or isotopically stable sources of water (for δ^18^O_en_; e.g., perennial lakes, rivers, or springs) and/or food (for δ^13^C_en_). The relatively large isotopic shifts over short time spans (within a few months of tooth growth) observed in some teeth (Figs 7d, 7h, and 8b) are best explained by dispersals or nomadic movements.

Another common pattern observed in portions of these teeth is an anticorrelation between δ^18^O_en_ and δ^13^C_en_ values when both display sinusoidal variation (Figs 7b, 7d, 7i-7j, and 8b). Interpreting δ^18^O_en_ as a seasonal indicator [30,84] and δ^13^C_en_ as reflecting precipitation amount [46] suggests wet summers and dry winters. This is consistent with patterns seen in summer monsoon regions [165]. Notably, this pattern is less common in mainland mammoths, likely due to higher mobility and exposure to more stable environmental or different climatic regimes. One mainland mammoth tooth (LACM HC 3805-L-12) (Fig 8b) shows δ^18^O_en_ and δ^13^C_en_ values that alternate between correlation and anticorrelation along its length, suggesting movement between regions with warm- and cold-season rainfall. Another mainland mammoth tooth (LACM HC 68190) records a pronounced mid-summer dip in δ^18^O_en_ values (Fig 8d) consistent with the amount effect during warm season precipitation [166]. Although it is unclear whether RLB had warm season precipitation or if these signals reflect migratory behavior, the serial δ^18^O_en_ data from these teeth indicate that some RLB mammoths were exposed to such climates. The anticorrelation between δ^18^O_en_ and δ^13^C_en_ values in several serially sampled NCI mammoth teeth suggests similar conditions for NCI mammoths.

## 4. Discussion

### 4.1. Reconciling enamel isotopes with the pollen record of southern California

The carbon isotope data indicate that the majority of mammoths living in Late Pleistocene southern California (both the NCI and RLB) consumed diets composed primarily of C_3_ vegetation (Figs 4 and 5). The slightly elevated mean δ^13^C_diet:meq_ value of −24.3‰ for RLB mammoths suggests that most individuals from this locality consumed either more water-stressed C_3_ plants or small amounts of C_4_ or CAM plants (Figs 4 and 5). A few individual mammoths—LACM/CIT 178, ORR 11, and LACM HC 68184— had δ^13^C_diet:meq_ values > −23‰, indicating that these mammoths either consumed a mixture of C_3_ and C_4_ plants, ingested CAM plants, and/or fed predominantly on material from trees experiencing severe water stress (likely of the family Cupressaceae and/or the genus *Pinus*) (Fig 4).

Macrofloral fossils [104,167] and pollen records [25,26,103,107] from southern California indicate the predominance of woodland/forest habitat—primarily *Pinus* and *Cupressaceae*—from ~60 ka to ~10 ka. The carbon isotope data show that *M. columbi* had a higher mean δ^13^C_diet:meq_ value (−24.3‰) than *M. exilis* (−26.5‰), suggesting drier and more open environments for *M. columbi*. These mean δ^13^C_diet:meq_ values for NCI and RLB appear to be consistent with microwear analysis results [28–29], which suggest that *M. exilis* browsed on more woody or leafy tree material, while *M. columbi* switched between browsing and grazing. Elevated δ^13^C_en_ values observed in one *M. columbi* tooth and in two *M. exilis* teeth (LACM HC 68184, LACM/CIT 178, and ORR 11) (Figs 4 and 5) may be explained by consumption of water-stressed pines or junipers, which exhibit higher δ^13^C values [46,49,50]. However, this interpretation is inconsistent with the microwear evidence if the water-stressed woody plants are assumed to be the cause of these higher δ^13^C_en_ values. The alternative proposed explanations—the consumption of C_4_ or CAM plants—are thus more likely, as they align with both the isotope data and microwear results.

While C_4_ grasses are not currently common in Southern California [45,81], due to a lack of sufficient summer rainfall [168], pollen records suggest that herbs are between ~30% and ~60% of the biomass in Southern California from 60 ka to today [103] and that grasses increased in some areas during warm and dry intervals [26]. Grass pollen cannot be used to distinguish C_3_ from C_4_ grass, thus we cannot exclude the possibility that C_4_ grasses and/or other C_4_ plants such as sedge or saltbush (*Atriplex* spp.) were present during the last glacial period in the region. In the modern climate, the North American Monsoon (NAM) brings moisture from the Gulf of California and Gulf of Mexico into northwestern Mexico and large areas of the southwestern United States, producing thunderstorms during the summer months [169]. California lies west of the core NAM region—receiving only occasional monsoon-related summer rainfall—and most modern precipitation in southern California occurs in winter and spring from westerly storms (Fig 2b) [84,170]. However, a potential strengthening of the NAM in the past, under a different climatic regime, could conceivably have brought sufficient summer rainfall to allow C_4_ grass expansion in the region. Uncertainties in the Late Pleistocene climate modeling [171] unfortunately leave some ambiguity as to which type of grass would have predominated in the region at that time. However, the apparent amount effect recorded in the δ^18^O_en_ values of LACM HC 68190 does seem to lend support to the idea that NAM was active in the study region during at least part of the Late Pleistocene.

CAM plants can also not be excluded as a possibility, especially for the RLB mammoths, which seem to have undertaken migrations or dispersals over large distances, similar to modern elephants [164]. For the NCI, CAM plants may be a possibility, but the evidence is less clear. The pollen records from the Santa Barbara channel [103] and from lake records in southern California [26] are based on the lowest taxonomic level identifications possible. Heusser (1998) notes various chaparral species (e.g., *Ceanothus*, *Adenostoma*, *Rhus*), none of which are expected to employ CAM photosynthesis. Modern chaparral flora in southern California is dominated by chamise (*Adenostoma fasciculatum*), scrub oak (*Quercus berberidifolia*), ceanothus (*Ceanothus* spp.), and manzanita (*Arctostaphylos* spp.) [172], though cacti (such as *Bergerocactus*) can also occur [172]. Chaparral pollen generally makes up less than 10% of the pollen record until ~14 ka [103], but it is present throughout the last glacial period. *Isoëtes*, an aquatic CAM plant [53,83], occurs sporadically at low abundances (~5% or lower) in southern California throughout the last glacial period [26]. Asteraceae make up large portions of the southern Californian pollen record [26] at certain times during the last glacial period, but which genera or species is not confirmed. Some genera in Asteraceae use CAM photosynthesis (such as *Senecio*) [83]. The lack of species-level identifications on some of the pollen record leaves open the possibility of CAM plants—at least in small proportions—on the island and/or in southern California, and these CAM plants could possibly account for the higher δ^13^C_en_ values of LACM/CIT 178, ORR 11, and LACM HC 68184.

### 4.2. Comparison of mammoths’ diets and environments between NCI and RLB

There are clear differences in both δ^13^C_en_ and δ^18^O_en_ values between NCI and RLB, reflecting differences in diet and environment (Fig 5). While the provenance of the SB mammoth ORR 6 is unknown, it is likely that ORR 6 was recovered from the NCI, given the similarity in δ^13^C_en_ and δ^18^O_en_ values between ORR 6 and the NCI mammoths (Table 1; Fig 5). ORR 6 remains excluded from NCI means to avoid introducing potential error; however, its inferred diet and environmental conditions overlap with those of the NCI mammoths and differ from those of the RLB mammoths. The reconstructed δ^13^C_diet:meq_ values (−26.4 ± 1.9‰) for NCI mammoths are, on average, lower than those for RLB mammoths (−24.2 ± 1.4‰). In contrast, both the MAP estimates (544 mm/y) and the reconstructed local water δ^18^O values for NCI (−5.5 ± 1.0‰) are on average higher than those for RLB (121 mm/y, −7.1 ± 0.9‰) (Fig 5; Tables 1 and 2). These differences most likely reflect temporal variation in the hydroclimate experienced by populations at these localities, given the very similar modern climatic conditions at both sites.

Integrating the δ^13^C-based MAP estimates with δ^18^O-based inferences of hydroclimate and temperature, the data suggest that NCI mammoths lived under relatively warmer and wetter conditions than RLB mammoths (Fig 5), likely during an interstadial, whereas the RLB population experienced a colder, drier stadial climate. The outliers—two pygmy mammoths (LACM/CIT 178 and ORR 11) from NCI and one Columbian mammoth (LACM HC 68184) from RLB (Fig 5)—all of which consumed either significant amounts of C₄ and/or CAM, or water-stressed C₃ woody plants—suggest dry, warm conditions, possibly reflecting episodes of enhanced aridity or increased summer rainfall during interstadials as C_4_ grasses require sufficient summer rain to grow [168].

### 4.3. Hydroclimate in the Late Pleistocene and the present

Both NCI and RLB have a semi-arid Mediterranean climate today, with annual rainfall concentrated in winter months (Fig 2). The estimated mean MAP for NCI (544 mm/yr), based on δ^13^C_diet:meq_ values, is higher than the modern average value (292.4 mm/yr) recorded at Anacapa Island (Western Regional Climate Center, http://www.wrcc.dri.edu), suggesting wetter conditions consistent with interstadials [113,114,116]. In contrast, the low mean estimated MAP (121 mm/yr) for RLB, based on δ^13^C_diet:meq_ values, is lower than the modern mean value (334.0 mm/yr) recorded in Culver City (Western Regional Climate Center, http://www.wrcc.dri.edu), suggesting that the area may have been even more arid than today consistent with stadials [113,114,116]. This interpretation, however, is inconsistent with the reconstructed δ^18^O_water:meq_ values for RLB, which suggest wetter conditions in the last glacial period compared to today (Fig 5), assuming that the relationships between monthly δ^18^O_weighted.precip_ values and precipitation amount and temperature during the late Pleistocene were similar to those of today, and that moisture sources remained relatively constant during the timespan of the studied mammoths. The low MAP estimates derived from RLB δ^13^C_diet:meq_ values may underestimate the local MAP due to the inclusion of C_4_ or CAM in the diet. This is supported by some of the serial samples from LACM HC 68575, which include δ^13^C_en_ values ≥ −8‰ (Fig 8e), indicating dietary shifts involving significant (~14–37%) contributions of CAM or C_4_ plants (S3 Table). LACM HC 3804-R-8 and LACM HC 3805-L-12 also have δ^13^C_en_ values approaching this threshold, along with high intra-tooth variability (Figs 8a and 8b), suggesting smaller proportions of C_4_ or CAM plant consumption (~6–24%) (S3 Table). If the higher-end MAP estimate from LACM HC 68579 (387 mm/yr) is compared with the modern MAP in the area (334 mm/yr), rainfall was, on average, 15% greater during the lifespan of this individual, possibly reflecting temporal or locality differences between this mammoth and other RLB mammoths.

### 4.4. Regional comparison of stable isotopes of Columbian mammoths

**Fig 9:**
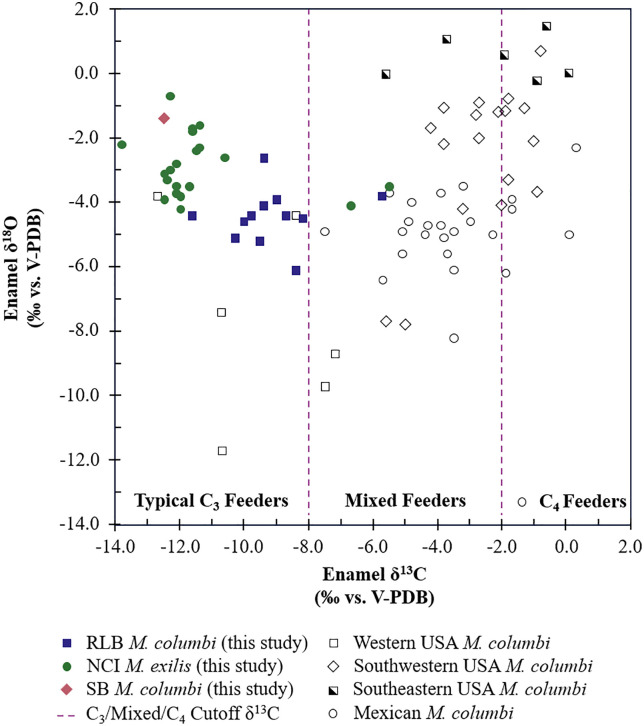
Enamelδ^13^C and δ^18^O values of mammoths across southern North America. Measured mammoth enamel δ^13^C and δ^18^O values from this study (closed symbols) are plotted with those of published values for *Mammuthus columbi* in other regions of southern North America (open symbols). Western USA mammoths are Columbian mammoths from Nevada [37] and California, USA [37,41]. Southwestern USA mammoths are Columbian mammoths from Arizona [37], New Mexico [37,39], and Texas, USA [29,39,41]. Southeastern USA mammoths are Columbian mammoths from Florida, USA [38]. Mexican mammoths are Columbian mammoths from central Mexico, northeastern Mexico, southeastern Mexico, and Baja California Sur [173–174]. The dashed lines represent the cutoffs between a pure browsing/typical C_3_ plant diet, mixed feeding/water stressed C_3_ diet, and grazing/C_4_ plant diet expected in enamel. These cutoffs assume an atmospheric correction of −0.94‰ between 0.06 and 0.01 Ma, an enrichment of +14.1‰ between diet and enamel for proboscideans, and modern plant carbon isotope composition ranges described in section 1.1. The means of serial samples were used as approximate bulk values to include with measured values from bulk samples.

Comparing the isotopic data for NCI and RLB mammoths with previous isotopic analyses of Columbian mammoths across southern North America [29,37–39,41,173,174] reveals that *M. exilis* and *M. columbi* from Santarosae appear to differ in diet from the eastern and central continental North American *M. columbi*, but are reasonably similar in diet to other sampled mammoths from western continental North America (Fig 9). The δ^18^O_en_ value ranges of NCI and RLB mammoths overlap with western continental North America *M. columbi*; however, within the western USA, samples from localities in Nevada have lower δ^18^O_en_ values than those from California mammoths, likely accounted for by the altitude effect and continental effect. The three sampled mammoths with higher δ^13^C_en_ values—LACM/CIT 178, ORR 11, and LACM HC 68184—have δ^13^C_en_ and δ^18^O_en_ values consistent with Mexican Columbian mammoths supporting that environmental conditions for these mammoths were warmer and drier, and that these mammoths might have consumed C_4_ and/or CAM plants.

## 5. Conclusions

Enamel δ^13^C values indicate that mammoths on Santarosae, like those on the southern California mainland, primarily consumed C_3_ plants, although whether this consisted of grasses, herbs, shrubs, and/or trees remains unclear. However, two NCI mammoths and one RLB Columbian mammoth have elevated enamel δ^13^C values (≥ −6.7‰), indicating a diet that included a mixture of C_3_ and C_4_ plants (ca. 13 to 46% C_4_), CAM plants, or woody plants under water stress. These outliers likely represent brief intervals of C_4_ plant emergence, CAM plant consumption, and/or the consumption of woody plants experiencing severe water stress. Most *M. columbi* from RLB have higher enamel δ^13^C values and lower δ^18^O values than those of Santarosae and coastal mammoths. However, overlap in enamel isotopic values between some *M. columbi* from RLB and NCI mammoths suggests that environmental conditions at the two sites were similar during certain intervals in the Late Pleistocene. This, along with very similar modern climatic conditions at these sites, implies that observed isotopic differences between the two localities most likely reflect temporal (i.e., stadial vs interstadial) variations, rather than spatial variations. Both the δ^13^C-based MAP estimates and reconstructed local water δ^18^O values for NCI suggest a wetter climate during the late Pleistocene than today. In contrast, the δ^13^C-based MAP estimates for RLB suggest a much drier late Pleistocene climate than today. However, this low estimate is likely due to consumption of small amounts of C_4_ and/or CAM plants by many of the *M. columbi* analyzed from RLB. Integrating the δ^13^C-based MAP estimates with δ^18^O-based inferences of hydroclimate and temperature, the data suggest that most NCI mammoths analyzed in this study lived under relatively warmer and wetter conditions than most RLB mammoths, likely during an interstadial, whereas the RLB population lived under colder and drier stadial conditions. The presence of C_4_, CAM, and/or water-stressed woody plants in the diets of some of these mammoths suggests that this region likely experienced either (1) sufficient summer rainfall to support C_4_ plants at both sites or (2) severe water stress affecting both sites during the lifetimes of these specific mammoths. Future work should prioritize radiometric dating of additional fossils from both localities, including some of the specimens analyzed in this study, and expanding isotopic sampling. A refined chronology and expanded isotopic sampling are necessary to elucidate the relationship between Late Pleistocene climate changes and the evolution and extinction of mammoths in the study region.

## Supporting information

S1 Fig Comparison of reconstruction methods using generalized relationships across taxa or elephant-specific relationships. The upper panel compares the reconstructed dietary vegetation isotope compositions (δ^13^C_diet:meq-BM_) —using the enrichment factor for Columbian mammoths [61] and the enrichment factor for pygmy mammoths calculated using the relationship from Tejada-Lara et al. (2018)—with the reconstructed dietary vegetation isotope compositions (δ^13^C_diet:meq_)—using enrichment factors from modern elephants. The lower panel compares the reconstructed water isotope compositions (δ^18^O_water, general_)—using a generalized equation for multiple taxa [72]—with δ^18^O_water_ calculated using a specific equation for modern elephants [69]. Applying these different methods would noticeably shift δ^13^C_diet:meq_ values of each species/locality toward one another (reducing separation by 1.3‰). The shift in δ^18^O_water_ values is small (only up to 0.23‰) relative to the 1.4‰ difference between locality means. However, these changes would not change the conclusions of (1) differing levels of rainfall for RLB and NCI, (2) the presence of three individuals with significant amounts of C_4_, CAM, or water-stressed conifer in the diet, or (3) that δ^18^O_water:meq_ values from RLB are lower than the modern mean δ^18^O_weighted.precip_ value and that NCI values are higher than the modern annual mean δ^18^O_weighted.precip_ value and lower than the modern unweighted annual mean δ^18^O_precip_ value.
(TIF)

S2 Fig Comparison of bulk and serial sampling. Enamel carbon and oxygen isotope ratios (δ^13^C_en_ and δ^18^O_en_) of bulk sampled teeth and the means of serially sampled teeth are plotted for both the Northern Channel Islands (NCI) and Rancho La Brea (RLB). Closed symbols show mean δ^13^C_en_ and δ^18^O_en_ values of serial samples while open symbols show δ^13^C_en_ and δ^18^O_en_ values of bulk samples. Mean δ^13^C_en_ values of each locality (µ δ^13^C_en_) for each sampling method are marked on the upper axis and mean δ^18^O_en_ values of each locality (µ δ^18^O_en_) for each sampling method are marked on the right axis. The three teeth with δ^13^C_en_ > −7.0‰. One standard deviation (σ) from locality means (µ) are outlined with dotted or dashed lines, with darker colored lines showing locality means for bulk sampled teeth and lighter colored lines showing locality means for serially sampled teeth. The mean differences between serially sampled and bulk sampled teeth from each locality (absolute difference in δ^13^C and δ^18^O values of 0.5‰ and 0.6‰ for the NCI and 0.4‰ and 0.3‰ for RLB) are much smaller than mean differences between localities (absolute difference in δ^13^C and δ^18^O values of 2.1‰ and 1.4‰). Mean differences between bulk samples and means of serial samples from each locality are similar to the differences measured in LACM HC 68190 (absolute difference in δ^13^C and δ^18^O values of 0.6‰ and 0.3‰), and so likely reflect the systematic differences caused by sampling method [136–138].
(TIF)

S1 Table Descriptions of mammoth teeth. (XLSX)

S2 Table δ^13^C and δ^18^O values of enamel samples analyzed in this study. (XLSX)

S3 Table Reconstructed modern-equivalent diet δ^13^C and water δ^18^O values. (XLSX)

S4 Table Relative abundances of various plant groups present in the pollen record and their modern equivalent δ^13^C values. (XLSX)

S5 Table Minimum, maximum, and range of serial sample δ^13^C and δ^18^O values. (XLSX)
