# Supplementary figures and images for: Diets and environments of late pleistocene pygmy and Columbian mammoths: Isotopic evidence from Southern California
Source: PLoS One. 2026 Jan 7;21(1):e0338674. doi: 10.1371/journal.pone.0338674 (PMC12779040; doi:10.1371/journal.pone.0338674)

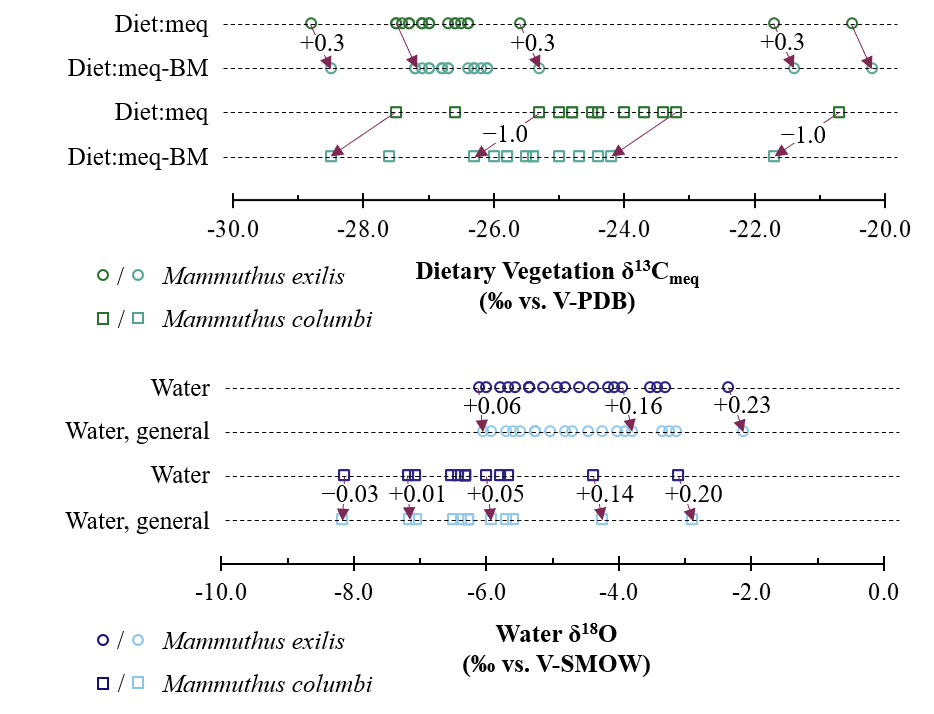

Supplement: S1 Fig — The upper panel compares the reconstructed dietary vegetation isotope compositions (δ13Cdiet:meq-BM) —using the enrichment factor for Columbian mammoths [61] and the enrichment factor for pygmy mammoths calculated using the relationship from Tejada-Lara et al. (2018)—with the reconstructed dietary vegetation isotope compositions (δ13Cdiet:meq)—using enrichment factors from modern elephants. The lower panel compares the reconstructed water isotope compositions (δ18Owater, general)—using a generalized equation for multiple taxa [72]—with δ18Owater calculated using a specific equation for modern elephants [69]. Applying these different methods would noticeably shift δ13Cdiet:meq values of each species/locality toward one another (reducing separation by 1.3‰). The shift in δ18Owater values is small (only up to 0.23‰) relative to the 1.4‰ difference between locality means. However, these changes would not change the conclusions of (1) differing levels of rainfall for RLB and NCI, (2) the presence of three individuals with significant amounts of C4, CAM, or water-stressed conifer in the diet, or (3) that δ18Owater:meq values from RLB are lower than the modern mean δ18Oweighted.precip value and that NCI values are higher than the modern annual mean δ18Oweighted.precip value and lower than the modern unweighted annual mean δ18Oprecip value. (TIF) [file pone.0338674.s001.tif]

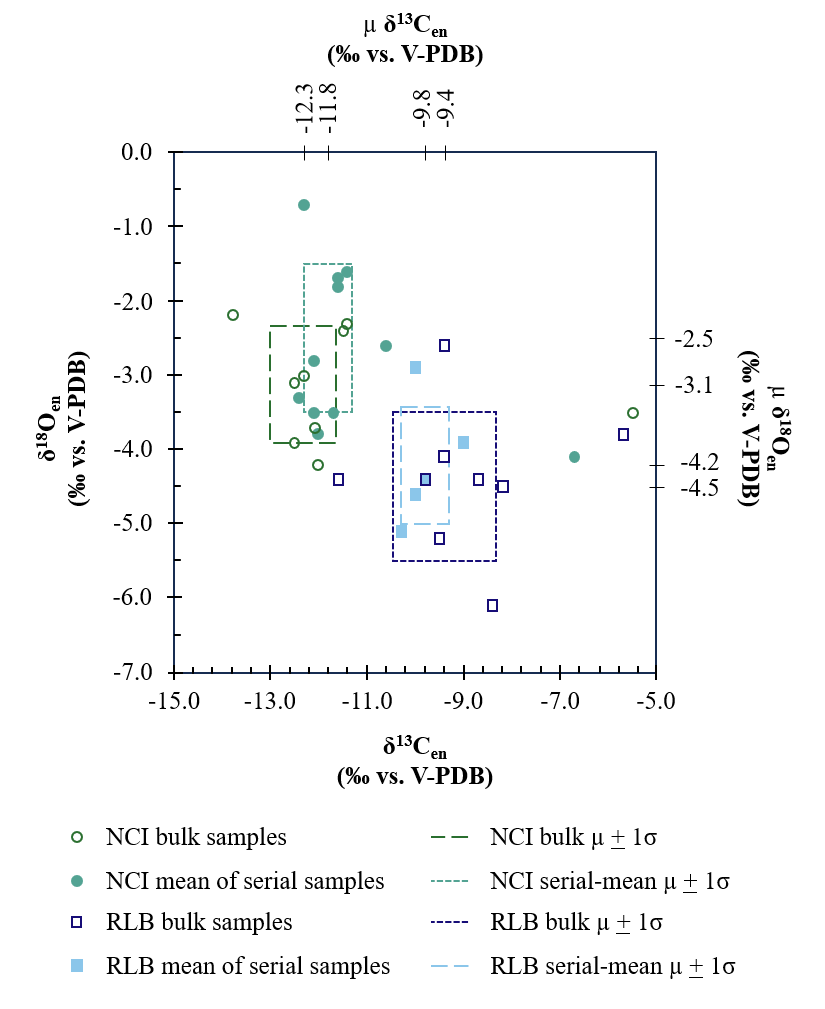

Supplement: S2 Fig — Enamel carbon and oxygen isotope ratios (δ13Cen and δ18Oen) of bulk sampled teeth and the means of serially sampled teeth are plotted for both the Northern Channel Islands (NCI) and Rancho La Brea (RLB). Closed symbols show mean δ13Cen and δ18Oen values of serial samples while open symbols show δ13Cen and δ18Oen values of bulk samples. Mean δ13Cen values of each locality (µ δ13Cen) for each sampling method are marked on the upper axis and mean δ18Oen values of each locality (µ δ18Oen) for each sampling method are marked on the right axis. The three teeth with δ13Cen > −7.0‰. One standard deviation (σ) from locality means (µ) are outlined with dotted or dashed lines, with darker colored lines showing locality means for bulk sampled teeth and lighter colored lines showing locality means for serially sampled teeth. The mean differences between serially sampled and bulk sampled teeth from each locality (absolute difference in δ13C and δ18O values of 0.5‰ and 0.6‰ for the NCI and 0.4‰ and 0.3‰ for RLB) are much smaller than mean differences between localities (absolute difference in δ13C and δ18O values of 2.1‰ and 1.4‰). Mean differences between bulk samples and means of serial samples from each locality are similar to the differences measured in LACM HC 68190 (absolute difference in δ13C and δ18O values of 0.6‰ and 0.3‰), and so likely reflect the systematic differences caused by sampling method [136–138]. (TIF) [file pone.0338674.s002.tif]
